# Anti-inflammatory effects of *Myrtus communis* L. (myrtle): experimental and clinical evidence with an immunological perspective

**DOI:** 10.1007/s10787-026-02173-x

**Published:** 2026-03-07

**Authors:** Ilhan Tahrali

**Affiliations:** Department of Pharmaceutical Microbiology, School of Pharmacy, Istanbul Medipol University, Goztepe Mah. Ataturk Cad. No: 40/16, 34815 Istanbul, Turkey

**Keywords:** *Myrtus communis* L., Medicinal plants, Inflammation, Anti-inflammatory, Immunomodulatory, Antioxidant

## Abstract

*Myrtus communis* L. (myrtle) is a medicinal plant widely used in traditional medicine. Due to the broad range of therapeutic applications of its leaves, fruits, and flowers, it has gained increasing scientific attention particularly in recent years in experimental models and clinical trials. Accumulating evidence highlights its anti-inflammatory properties, alongside well-documented antioxidant and antimicrobial effects, providing a scientific basis for its traditional use. This review provides a comprehensive and critical overview of the anti-inflammatory effects of *M. communis* based on in vitro, in vivo, and clinical studies. It systematically synthesizes the literature from past to present, while highlighting methodological limitations and unresolved gaps, particularly from an immunological perspective, with the aim of guiding future ethnopharmacological and translational research on *M. communis*. The plant parts utilized, extraction methods, routes of administration, and the experimental and clinical models employed in studies indexed in PubMed were systematically analyzed and compared. Regardless of these variables, the available evidence consistently demonstrates that myrtle exerts suppressive and preventive effects on inflammatory responses. However, the evaluation of inflammation in most studies has predominantly relied on histopathological assessments and indirect demonstration of anti-inflammatory activity through the measurement of antioxidant-related parameters. In contrast, investigations directly addressing key components of the immune system that play central roles in inflammatory processes remain relatively limited. Future studies employing standardized experimental approaches and incorporating immune-related endpoints are required to elucidate the molecular and cellular mechanisms underlying the anti-inflammatory effects of *M. communis.*

## Introduction

*Myrtus communis* L. (myrtle), a member of the Myrtaceae family, is an aromatic medicinal plant in evergreen shrub or small tree form (Nedjimi 2024). It is distributed primarily in the coastal areas of Mediterranean countries including North Africa and Southern Europe, as well as in the tropical regions of Australia, and the Middle Eastern countries (Al-Snafi et al. 2024). It is a 1–3 m tall perennial plant with small, grape-like, usually purple-black fruits, 2–5 cm long evergreen leaves and white-pinkish fragrant flowers (Gencler-Ozkan and Gencler-Guray 2009).

*M. communis* contains a wide range of bioactive compounds, including polyphenols, flavonoids, tannins, quinones, anthraquinones, anthocyanins, saponins, and vitamins (Asgarpanah and Ariamanesh 2015). Although there are numerous scientific studies on its composition and biological activities, most chemical analyses have focused on the leaves and leaf-derived essential oils (EOs). However, the fruits of *M. communis* are also valuable for their fatty acid content and richness in volatile oils, sugars, tannins, organic acids, and flavonoids (Asgarpanah and Ariamanesh 2015; Yasa et al. 2023). The main chemical constituents of *M. communis* are summarized in Table 1.

**Table 1:** Major phytochemical classes and representative compounds reported in different parts of *Myrtus communis* L.

Plant part	Main groups	Subclass	Key compounds	References
Leaves	Essential oils	Monoterpenes / oxygenated monoterpenes / phenylpropanoids	α-Pinene, limonene; 1,8-cineole, linalool, α-terpineol; myrtenyl acetate, geranyl acetate, linalyl acetate; methyl eugenol	Karimi et al. 2025; Yarahmadi et al. 2024; Barboucha et al. 2025;
	Phenolic compounds	Phenolic acids	Gallic acid, caffeic acid, syringic acid	Snoussi et al. 2021; Aidi Wannes et al. 2010
		Flavonoids	Quercetin, quercetin 3-*O*-glucoside, quercetin 3-*O*-rhamnoside, quercetin 3-*O*-galactoside, myricetin, myricetin 3-*O*-galactoside, myricetin 3-*O*-rhamnoside, kaempferol	
Fruits	Phenolic compounds	Phenolic acids and tannins	Gallic acid, ellagic acid, chlorogenic acid; hydrolysable tannins	Dessì et al. 2025; San et al. 2015
		Flavonoids	Hyperoside, quercitrin, quercetin 3-*O*-galactoside, quercetin 3-*O*-rhamnoside, quercetin 3-*O*-glucoside, myricetin glycosides, naringin, apigenin 7-*O*-glucoside	Dessì et al., Montoro et al. 2006
		Anthocyanins	Delphinidin 3-*O*-glucoside, petunidin 3-*O*-glucoside, malvidin 3-*O*-glucoside	Montoro et al. 2006
	Fixed oils	Fatty acids	Oleic acid, palmitic acid, stearic acid, linoleic acid	Qader et al. 2017; Sumbul et al. 2011
	Essential oils	Monoterpenes / oxygenated monoterpenes	α-Pinene, limonene; 1,8-cineole, linalool, α-terpineol, geraniol; myrtenyl acetate, α-terpinyl acetate	Cvitković et al. 2024; Usai et al. 2018
	Phytosterols	Sterols	β-Sitosterol, campesterol, Δ5-avenasterol, Δ7-sitosterol	Cvitković et al. 2024
Seeds	Fixed oils	Fatty acids	Linoleic acid, palmitic acid, oleic acid, stearic acid	Mezni et al. 2025; Al-Najjar et al. 2025; Wannes and Marzouk 2016
Flowers	Essential oils	Monoterpenes / phenylpropanoids	α-Pinene, α-terpinene; 1,8-cineole, linalool, α-terpineol; eugenol, methyl eugenol; geranyl acetate	Barhouchi et al. 2023

*M. communis* is widely used for its medicinal properties, with its leaves commonly consumed as tea and its edible fruits (Alipour et al. 2014). The EO and hydrosol obtained from the flowering herb parts by distillation are used in pharmaceutical products, cosmetics and perfumery. The fruits and leaves are also used in the food industry for the production of sweet liqueurs (Dabbaghi et al. 2023).

### Ethnopharmacological background of *Myrtus communis* L.

Various parts of *M. communis* are known to have been utilized in ancient medicine for a wide range of therapeutic purposes, including the treatment of respiratory and digestive system disorders, wounds and burn healing, management of oral lesions and toothaches, relief of insect bites, and prevention of inflammation (Alipour et al. 2014). In traditional medicine, *M. communis* has been widely used for the treatment of respiratory tract infections, stomachaches and other gastrointestinal disorders, diarrhea, prostate inflammation, and pyogenic infections. Moreover, it has been reported to possess analgesic, expectorant, antiseptic, anti-diabetic, anti-inflammatory, and wound-healing agent (Sen et al. 2016a).

Scientific research studies motivated by the long-standing use of *M. communis* have shown that, in addition to its antioxidant, antibacterial and antifungal effects (Chalchat et al. 2010; Ozcan and Boyraz 2000; Ozcan et al. 2015; Sagdic and Ozcan 2003), it can be used in wound treatment due to its astringent, tonic and antiseptic properties, as well as digestive and urinary system disorders (Alipour et al. 2014; Sisay and Gashaw 2017). Due to its well-documented antimicrobial properties, numerous dermocosmetic formulations containing *M. communis* leaf extracts have been developed and commercialized. However, despite its widespread and long-standing use in traditional medicine, *M. communis* has not yet been incorporated into regulatory-approved prescription pharmaceutical formulations for therapeutic purposes.

Building on its therapeutic properties, extensive research has highlighted the immunomodulatory potential of *M. communis*, particularly its anti-inflammatory activities. Among the various studies examining the anti-inflammatory properties of *Myrtus communis* L., the majority consist of experiments in which anti-inflammatory activity is primarily assessed through macroscopic observations and biochemical evaluations. The aim of this review is to present a comprehensive analysis of research on the anti-inflammatory effects of *M. communis.* To this end, a brief overview of inflammation is presented, followed by in vivo, in vitro, and clinical studies on the anti-inflammatory properties of *M. communis*. The literature discussed in the review was identified through a comprehensive search of the PubMed database, covering studies from the earliest available reports to the most recent publications.

### Inflammation and inflammatory mediators

Inflammation is the immune system’s protective response to detrimental agents, such as pathogens, damaged cells, toxic compounds and radioactive rays. It is a vital defense mechanism that initiates the elimination of harmful stimuli and promotes tissue healing (Chen et al. 2017). As a complex physiological process, inflammation is characterized by the activation of immune cells, the release of molecular and cellular mediators, and alterations in vascular function. During inflammatory responses, proinflammatory cytokines, chemokines, and other signaling molecules establish a communication network in response to cellular damage caused by internal or external factors. The alterations in vascular permeability result in the migration and accumulation of immune cells and plasma proteins to recruit and accumulate at the site of inflammation, eliminate inflammatory agents, and initiate tissue repair (Chavda et al. 2024).

Various stimuli trigger the inflammatory process by activating innate immune cells through pattern recognition receptors (PRRs), such as Toll-like receptors (TLRs), which transmit signals that activate transcription factors like nuclear factor-kappa B (NF-κB). This signaling enables innate immune cells, including dendritic cells, neutrophils, macrophages, mast cells, and other tissue-resident cells, to recognize inflammatory stimuli and release mediators such as cytokines and chemokines that drive the inflammatory response. Collectively, these immune cells and soluble components orchestrate the initiation, maintenance, and regulation of inflammation (Hanada and Yoshimura 2002).

The type and magnitude of the inflammatory response are determined by the causative factor, such as infectious agents (viruses, bacteria or parasites) and tissue injury (Medzhitov 2010). In bacterial infections, tissue-resident macrophages are activated via their receptors (e.g., TLRs), leading to the secretion of inflammatory cytokines (IL-1β, IL-6, TNF-α), chemokines, and proinflammatory lipid mediators (e.g., prostaglandins). In the case of viral infections, natural killer (NK) cells and cytotoxic lymphocytes releasing IFN-α and IFN-β are activated, whereas parasitic infections stimulate basophils and mast cells, resulting in the production of IL-4, IL-5, and IL-13 (Freire and Van Dyke 2013).

Acute inflammation begins rapidly, within minutes or hours, and is resolved within a few days. The process typically involves neutrophil-dominated cellular infiltration, followed by erythema and edema, which arise from increased vascular permeability and elevated blood flow to the inflamed area due to vasodilation (Hannoodee and Nasuruddin 2025; Abdulkhaleq et al. 2018). Beyond immune cells, transcription factors, cytokines, and chemokines, the inflammatory process is also mediated by reactive oxygen species (ROS), reactive nitrogen species (RNS), acute-phase proteins such as C-reactive protein (CRP), prostaglandins, cyclooxygenase (COX)-derived metabolites, and growth factors associated with inflammation (Stone et al. 2025). When acute inflammation cannot be eliminated for more than six weeks, it may progress to chronic inflammation, characterized by a sustained and dysregulated inflammatory state (Furman et al. 2019). In this context, the adaptive immune system—primarily T lymphocytes—plays a pivotal role by producing pro-inflammatory cytokines that maintain and intensify the inflammatory response, although innate immune components may also contribute (Moro-García et al. 2018).

Since untreated acute inflammation may result in severe complications, including tissue damage, systemic effects, or progression to chronic inflammation, improving the inflammatory condition and achieving complete resolution are of critical importance (Furman et al. 2019). Chronic inflammation, in turn, plays a central role in the pathogenesis of numerous non-communicable diseases, including cardiovascular diseases, obesity, arthritis, diabetes, cancer, and autoimmune disorders (Libby 2007). This underscores the need to investigate inflammatory mechanisms and the cellular and soluble components implicated in the pathogenesis of a wide range of diseases.

The treatment of inflammation is determined by the underlying cause (e.g., infection or trauma), the nature of the inflammatory response (acute or chronic), and its severity. The main pharmacological agents employed in conventional treatment include glucocorticoids and nonsteroidal anti-inflammatory drugs (NSAIDs). Glucocorticoids suppress the synthesis of prostaglandins and inflammatory proteins, while NSAIDs exert their effects by inhibiting the cyclooxygenase (COX) enzyme to reduce the production of inflammatory mediators (Nunes et al. 2020). NSAIDs, employed in the management of acute and chronic inflammation, are among the most widely prescribed medications worldwide (Virshette et al. 2019).

Although conventional medications used to suppress and eliminate inflammation are often efficacious, they may cause serious adverse effects and do not always ensure complete resolution (Ghasemian et al. 2016). As a result of increased search for safer and more effective anti-inflammatory agents, interest in medicinal plants has grown markedly, due to their promising anti-inflammatory potential, largely attributed to secondary metabolites such as polyphenols (Owona et al. 2020). The ability of medicinal plants to suppress and resolve inflammation has long been recognized in traditional and folk medicine. Nevertheless, the safe and rational use of these plants requires a clear understanding of their pharmacological properties and scientific confirmation of their efficacy. In this context, the present review provides a comprehensive perspective on the potential role of *Myrtus communis* L. in modulating inflammatory responses.

## Anti-inflammatory effects of *M. communis*

**Fig. 1:**
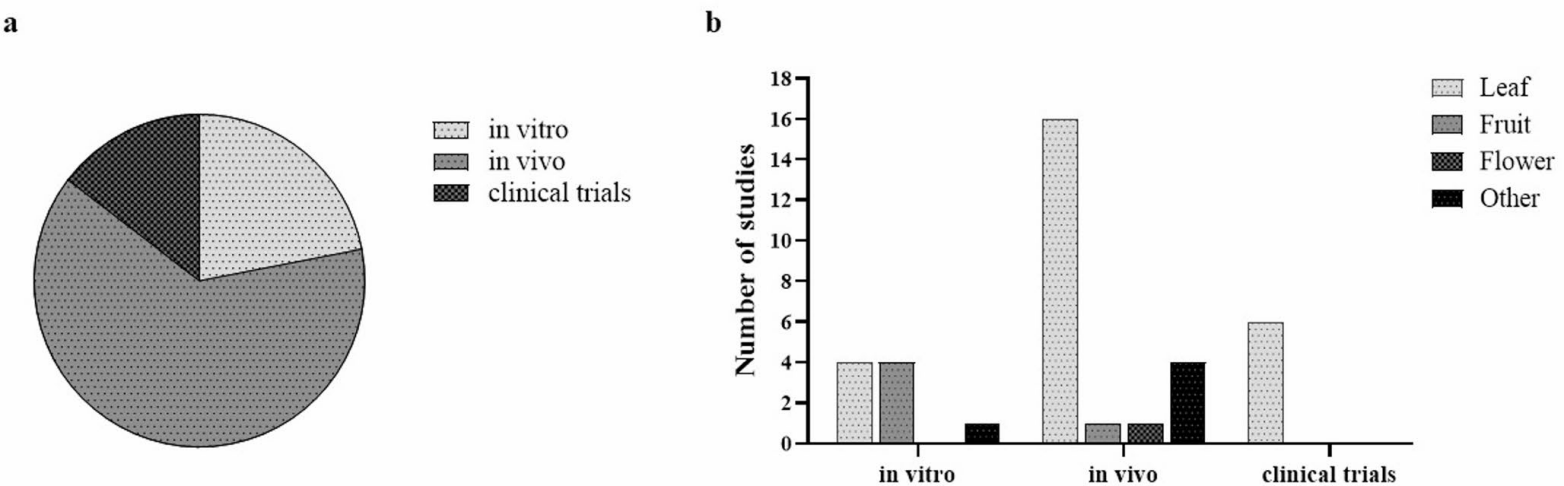
Classification of studies evaluating the anti-inflammatory activity of *Myrtus communis* L. according to study type and plant parts used. **a** Distribution of in vitro, in vivo, and clinical studies conducted with *M. communis*; **b** Distribution of investigated plant parts across experimental models

The widespread use of plants in traditional and folk medicine to treat conditions now recognized as inflammatory disorders has provided contemporary medical practice and scientific research. *M. communis* is among the medicinal plants currently under investigation for its antimicrobial, antioxidant, anticancer, and potential anti-inflammatory effects. Research on its anti-inflammatory properties has predominantly relied on in vivo animal models, followed by in vitro experiments (Fig. 1a). More recently, especially within the past five years, clinical trials have also gained momentum. Although the majority of these studies employed the leaves of the *M. communis*, a limited number of investigations also examined its fruits, flowers, and seeds (Fig. 1b).

The following sections summarize findings from in vitro, in vivo, and clinical studies that have explored the potential role of *M. communis* in modulating inflammation. Table 2 summarizes the plant parts used, the extraction methods applied, and the experimental and clinical models investigated.

**Table 2:** Characteristics of studies investigating *Myrtus communis* L.

Study type	Plant part	Preparation / extract	Experimental model (species/cell line)	Inflammatory model /outcome	References
In vitro	Leaf	Methanol extract-derived MCA-1	THP1, J774.2, U138MG cells	Inflammatory pathways	Soomro et al. 2019
		EO (commercial)	RAW 264.7 cells	Macrophage activation	Leigh-de Rapper et al. 2021
		Lipophilic dry extract	Mo-DCs	*C. acnes*-induced inflammation	Mias et al. 2023a
		Aqueous propylene glycol extract	HaCaT keratinocytes	Inflammatory responses	Kilic et al. 2019
	Fruit	Aqueous extract	Human neutrophils	Apoptosis	Amira et al. 2012
		Aqueous ethanol extract	mBMMC	Mast cell degranulation	Zaidi et al. 2014
		Aqueous ethanol extract	AGS	*H. pylori*-induced inflammation	Zaidi et al. 2012
		Hydrodistillation (EO)	HUVECs	Sepsis-induced endothelial cell damage	Kutlu et al. 2021
	Pulp and seed waste	Aqueous ethanol extract	Human skin fibroblasts (HFF-1)	Oxidative stress and inflammation	Cruciani et al. 2019
In vivo	n.s	Ethanol extract	Rat	Paw edema	Al-Hindawi et al. 1989
	Leaf	Hydrodistillation (EO)	Wistar albino rat	Ear edema	Maxia et al. 2011
		Hydrodistillation (EO)	Wistar albino rat	Paw edema	Belahcene et al. 2023
		Methanol extract	Wistar albino rat	Paw edema	Belahcene et al. 2024
		Ethanol extract	Wistar albino rat	Burn wound	Ozcan et al. 2019
		Methanol extract	Wistar rat	Infected burn wound	Jafari et al. 2024
		Ethanol extract	Wistar albino rat	Acute pancreatitis	Ozbeyli et al. 2020
		Ethanol extract	Wistar albino rat	HFD-fed pancreas	Kabatas et al. 2024
		Hydrodistillation (EO)	Wistar rat	Acute gastric lesions	Mansour et al. 2022
		Ethanol extract	Wistar albino rat	Bile duct ligation	Sen et al. 2016b
		Ethanol extract	Wistar albino rat	Nephrolithiasis	Ertas et al. 2024
		Ethanol extract	Wistar albino rat	Alzheimer’s disease	Aykac et al. 2019
		Ethanol extract	Wistar albino rat	Renovascular hypertension	Cevikelli-Yakut et al. 2020
		Hydrodistillation (EO)	BALB/c mouse	Toxoplasmosis	Shaapan et al. 2021
		Methanol macerates	Albino rat	Pulmonary fibrosis	Samareh Fekri et al. 2018
		Hydrodistillation (EO)	Sprague Dawley	Healthy (non-inflamed) model	Odeh et al. 2022
		Ethanol extract	Wistar albino rat	Testicular damage	Coskunlu et al. 2024
	Aerial parts	Aqueous and ethanol extracts	Albino mouse	Ear edema and cotton pellet-induced granuloma	Hosseinzadeh et al. 2011
	Fruit	Aqueous extract	Mouse	Paw and ear edema	Amira et al. 2012
	Seed / stems	Methanol extract	Sprague–Dawley rat	Oral ulcer	Hashemipour et al. 2017
		Aqueous extract	Wistar rat	Esophagitis	Jabri et al. 2016
		Aqueous extract	Swiss albino mouse	Gastrointestinal nematode	Jabri et al. 2021
	Flower	Hydrodistillation (EO)	Wistar rat	Hepatotoxicity	Ben Hsouna et al. 2019
Clinical trial	Leaf	Boiled water extract	Adult patients	Recurrent aphthous stomatitis	Babaee et al. 2010
		Aqueous and ethanol extracts	Pediatric patients	Gingivitis	Mohamed-Ali et al. 2024
		Ethanol extract	Adult patients	Acne vulgaris	Salmanian et al. 2020
		Ethanol macerate	Adult patients	Gingivitis	Talebi Ardakani et al. 2022
		Ethanol extract	Adult patients	Acne vulgaris	Bagatin et al. 2023
		Ethanol extract	Pediatric and adult patients	Acne vulgaris	Mias et al. 2023b

n.s., not specified; EO, essential oil(s); HFD, high-fat diet; Mo-DCs, monocyte-derived dendritic cells; HUVECs, human umbilical vein endothelial cells; RAW 264.7, murine macrophage cell line; THP-1, human monocytic cell line; J774.2, murine macrophage cell line; U138MG, human glioblastoma cell line; HaCaT, human keratinocyte cell line; mBMMC, mouse bone marrow–derived mast cells; AGS, human gastric adenocarcinoma cell line; HFF-1, human foreskin fibroblast cell line; *C. acnes*, *Cutibacterium acnes*; *H. pylori*, *Helicobacter pylori*

### In vitro studies

**Fig. 2:**
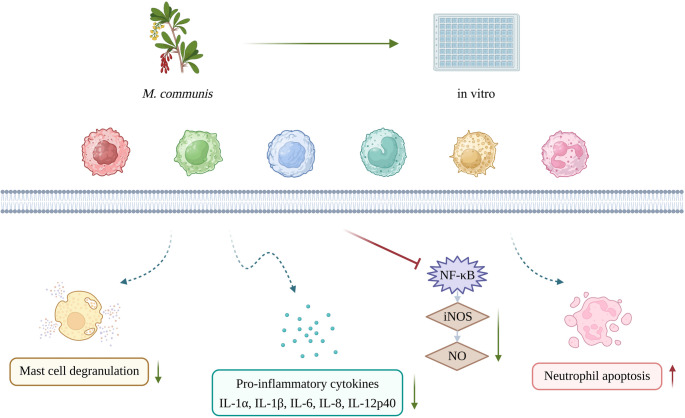
Schematic overview of the common anti-inflammatory effects of *Myrtus communis* L. reported in in vitro studies

In vitro studies investigating the anti-inflammatory properties of *M. communis* have emerged predominantly within the past 15 years, indicating that this line of research is relatively recent and remains under active development. In these studies, conducted on immune and non-immune cells, extracts and EOs derived predominantly from the leaves and fruits of myrtle were employed (Fig. 1b). The common findings obtained from in vitro studies on the effects of *M. communis* are schematically summarized in Fig. 2.

#### Immune cell-based models

Among the earliest in vitro investigations, the study by Amira et al. examined the apoptotic effects of an aqueous extract of myrtle fruits on human peripheral blood neutrophils. Myrtle fruits, along with lavender flowers, were found to reduce the viability of pro-inflammatory neutrophils, suggesting a potential suppressive role in acute inflammation (Amira et al. 2012).

*M. communis* fruits, along with various other medicinal plants, were also tested for their effects on mast cell degranulation. Mucosal-type mouse bone marrow-derived mast cells (mBMMCs) from BALB/c mice were pre-treated with aqueous ethanol extracts and then cultured following antigen- or ionophore-induced degranulation. Although the strongest effect was observed with *Alpinia galangal*, higher extract concentrations of the tested plants, including *M. communis,* were also found to suppress both IgE/antigen- and ionophore-induced degranulation in mBMMCs (Zaidi et al. 2014). These findings may indirectly indicate the anti-inflammatory potential of *M. communis*, as inhibition of degranulation limits the release of inflammatory mediators.

The effects of Myrtucommuacetalone 1 (MCA-1), isolated from the methanolic extract of myrtle leaves rather than the whole leaf extract, were analyzed in THP1 human monocytes, U138MG glioblastoma cells, and J774.2 mouse macrophages to assess its influence on the inflammatory response. MCA-1 was shown to inhibit LPS-induced nitric oxide (NO) production in THP1 cells in a dose-dependent manner, whereas no inhibitory effect was observed in U138MG cells. Activation of the transcription factors NF-κB and p38 kinase, which contribute to the production of pro-inflammatory cytokines, is crucial for the expression of inflammatory genes such as inducible nitric oxide synthase (iNOS). Although MCA-1 had no effect on p38 activation, increasing doses almost completely inhibited NF-κB translocation in J774.2 cells. Consistently, MCA-1 was also demonstrated to suppress iNOS mRNA expression and NF-κB phosphorylation in a dose-dependent manner, supporting its potential as an anti-inflammatory agent (Soomro et al. 2019).

In a study examining the antimicrobial and anti-inflammatory effects of 49 EOs commonly used in aromatherapy for respiratory conditions, including *M. communis*, NO production was measured in RAW 264.7 macrophages following LPS stimulation for 24 h in the presence of EO combinations. Among all tested combinations, *M. communis* in combination with *Melaleuca alternifolia*, together with 4 other EO combinations, demonstrated the most potent anti-inflammatory effects by suppressing NO production (Leigh-de Rapper et al. 2021).

In recent years, several studies have been conducted on the potential use of *M. communis* in the treatment of acne vulgaris, a chronic inflammatory skin disease. Mias et al. assessed the ability of *M. communis* and celastrol-enriched plant cell culture (CEE) extracts to regulate inflammation in *Cutibacterium acnes*-induced human monocyte-derived dendritic cells (Mo-DCs) in comparison with dexamethasone. The cells were stimulated with *C. acnes* and cultured for 24 h in the presence of ethanol extracts of myrtle leaves and CEE. Exposure to *C. acnes* led to a significant elevation in the levels of inflammatory cytokines IL-6, IL-8, IL-12p40, and TNF-α in the supernatants of Mo-DCs. Treatment with myrtle extract attenuated IL-6 and IL-12p40 levels, whereas CEE lowered TNF-α in addition to IL-6 and IL-12p40. Co-application of both agents resulted in a stronger anti-inflammatory response, notably reducing IL-8 and TNF-α levels, which remained unchanged when either agent was applied separately (Mias et al. 2023a).

#### Non-immune cell models

Building on the traditional use of myrtle fruit as an antidiarrheal, carminative, and cardiac tonic, Zaidi et al. investigated the anti-inflammatory property of *M. communis* on gastric epithelial cells infected with *Helicobacter pylori*. AGS human gastric cancer cells were pretreated with 24 plant extracts, including an aqueous-ethanol extract of myrtle fruits, and subsequently exposed to *H. pylori* for 4 h in the presence or absence of the extracts. Cells were also stimulated with TNF-α without *H. pylori* infection to evaluate the effects of the extracts on non-infectious or exogenously induced inflammatory responses. After the culture, myrtle, along with three other plants, demonstrated robust inhibitory activity on IL-8 secretion in *H. pylori*-infected cells. Although the strongest response was observed with *Cinnamomum cassia*, the findings indicated that myrtle may also help prevent *H. pylori*-induced inflammation (Zaidi et al. 2014).

Another study evaluating the anti-acne potential of *M. communis* tested two different extract mixtures on the human keratinocyte cell line (HaCaT cells). The first mixture consisted of aqueous propylene glycol extracts of *M. communis* leaves, *Juglans regia* husk, *Matricaria chamomilla* flowers, *Urtica dioica* leaves, and *Rosa damascene* flowers. The second mixture included aqueous extracts of *Brassica oleracea* var. *botrytis* and *Brassica oleracea* var. *italica*. Gene expression levels of IL-1α, and TNF-α were analyzed in HaCaT cells after treatment with the two extract mixtures. Whereas the second mixture reduced IL-1α and TNF-α levels, the myrtle-containing mixture significantly suppressed IL-1α but upregulated TNF-α expression. However, both mixtures exhibited strong anti-inflammatory effects, indicating their potential as candidates for topical acne treatment (Kilic et al. 2019).

In a study utilizing a distinct material—aqueous ethanol extracts of myrtle pulp and seeds obtained from liquor production by-products—the extracts were tested on human skin fibroblast 1 (HFF1) cells. Following 12–24 h of pre-treatment, cells were incubated with H_2_O_2_ for 1 h to induce oxidative stress. Treatment with both pulp and seed extracts significantly reduced H₂O₂-induced reactive oxygen species (ROS) in a dose-dependent manner. The elevated mRNA levels of IL-1β, IL-8 and TNF-α were also significantly decreased by treatment with both extracts, with the pulp extract exhibiting the strongest anti-inflammatory activity. Furthermore, VEGF-A gene expression, which is implicated in vascular inflammation, was downregulated by the treatment (Cruciani et al. 2019).

Unlike in vivo studies involving leaf extracts, in vitro studies with *M. communis* have predominantly focused on the anti-inflammatory properties of the fruits. In this regard, hydrodistilled EOs and compounds obtained from myrtle fruits were examined for their capacity to mitigate sepsis-induced endothelial cell damage in an in vitro model using human umbilical cord vein endothelial cells (HUVECs). HUVECs were exposed to LPS to induce a sepsis model, followed by treatment with the extracts, and mRNA levels of TNF-α, IL-1β, IL-6, and endothelial nitric oxide synthase (eNOS) were then measured. While α-terpineol and EO treatment failed to mitigate the cellular damage resulting from LPS-induced elevation of eNOS, IL-1β, IL-6, and TNF-α mRNA expression, treatment with both 1.8-cineole and α-pinene markedly suppressed these pro-inflammatory mediators (Kutlu et al. 2021).

### In vivo studies with *M. communis*

**Fig. 3:**
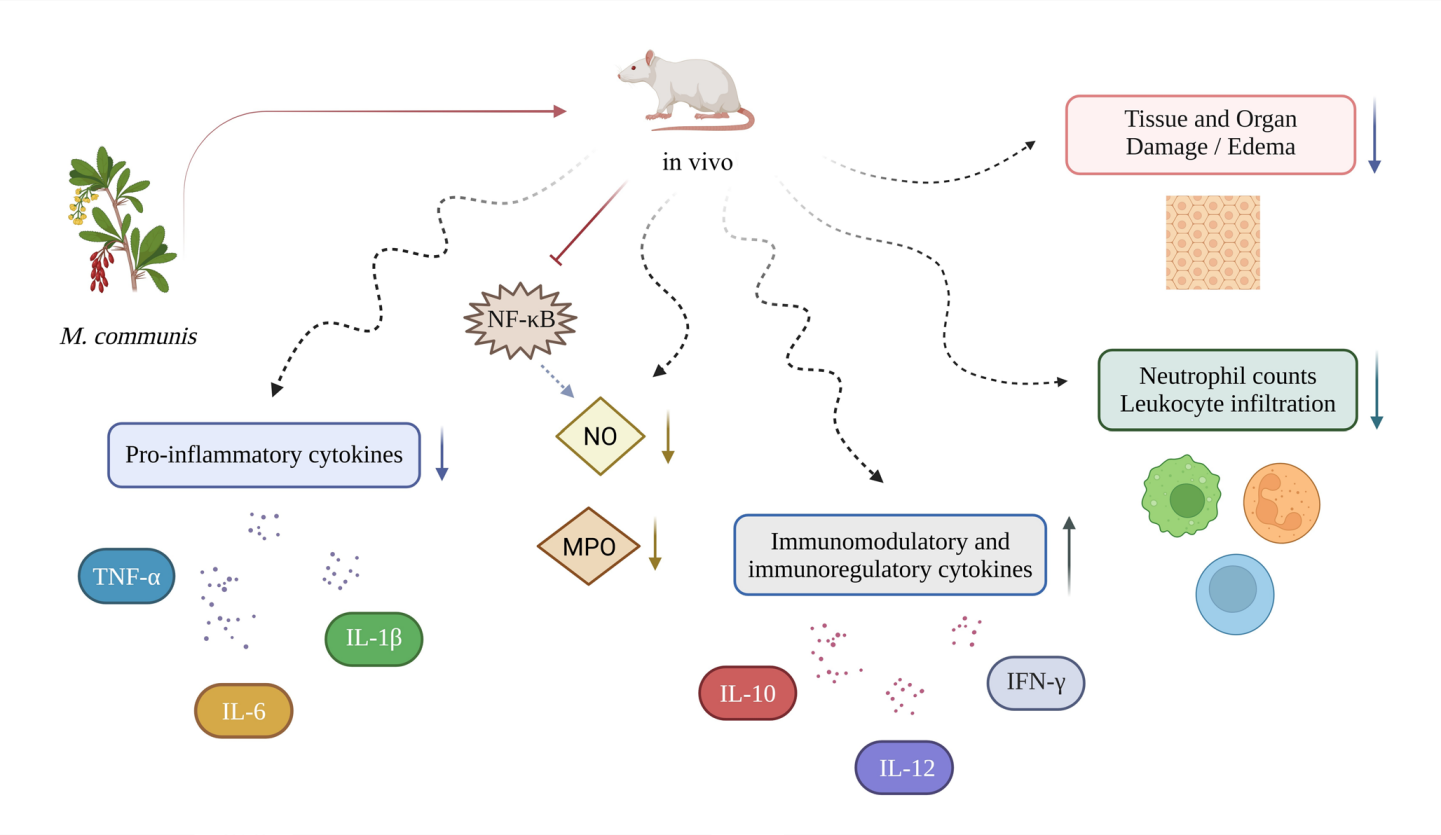
Schematic overview of the common anti-inflammatory effects of *Myrtus communis* L. observed in in vivo studies

The anti-inflammatory effects of *M. communis* have been investigated more comprehensively in vivo than in vitro. In vivo studies investigating the anti-inflammatory potential of *M. communis* have predominantly assessed biochemical markers of oxidative stress and the antioxidant defense system, including malondialdehyde (MDA), glutathione (GSH), superoxide dismutase (SOD), catalase (CAT), and glutathione peroxidase (GPx), because of their close association with inflammatory processes. Nevertheless, these molecules do not serve as direct inflammatory markers; instead, their modulation offers indirect insights into the anti-inflammatory potential of the investigated agents. On the other hand, direct evidence regarding the anti-inflammatory effects of *M. communis* has been derived mainly from clinical observations and histopathological analyses, whereas only a limited number of animal studies have assessed inflammatory cytokine profiles, which have a key role in the inflammatory response, thereby providing more direct mechanistic insights. Among in vivo investigations, several have employed acute inflammation models to evaluate the rapid effects of *M. communis*, while others have examined chronic and organ-specific models, as outlined below. The common anti-inflammatory mechanisms of *M. communis* identified across in vivo models are schematically summarized in Fig. 3.

#### Acute and chronic inflammation models

As one of the most prominent signs of inflammation, edema serves as a useful parameter for evaluating the anti-inflammatory effects of pharmacological or natural agents. Accordingly, anti-edema activity is commonly assessed in studies investigating the anti-inflammatory potential of medicinal plants. Among experimental animal models of acute inflammation, the carrageenan-induced paw edema model, as well as TPA- or xylene-induced ear edema models, are frequently employed.

Carrageenan is a well-established pro-inflammatory agent commonly used to induce acute inflammatory responses, resulting in the development of paw edema (Belahcene et al. 2024). One of the earliest studies examining the anti-inflammatory effects of *M. communis* was conducted on rats with paw edema induced by carrageenan. Although the ethanol extract of *M. communis* was effective in reducing edema, its efficacy appeared to be weaker than that of *Apium graveolens*, *Matricaria chamomilla*, *Withania somnifera*, and *Achillea santolina* (Al-Hindawi et al. 1989). Building on earlier findings, Rossi et al. investigated the effects of Myrtucommulone (MC), a purified compound isolated from myrtle leaves, on carrageenan-induced paw edema in mice. Pretreatment of mice with increasing doses of MC was shown to reduce the inflammatory response by suppressing paw edema (Rossi et al. 2009).

In addition to the leaves, myrtle fruits have been investigated for their anti-inflammatory activity, as well as for their apoptotic effects on pro-inflammatory cells, using paw edema model. The aqueous extract of *M. communis* fruits was administered orally to mice prior to edema induction, and its effects were compared with those of *Calamintha nepeta, Lavandula stoechas* and *Smilax aspera* extracts. *M. communis* berries exhibited the highest inhibitory activity against carrageenan-induced paw edema, with 60% inhibition at 3 h. Moreover, only myrtle berries showed a significant inhibitory effect at 5 h, with 35% reduction. Interestingly, none of the extracts had any effect on the TPA-induced ear edema model (Amira et al. 2012).

Hosseinzadeh et al. investigated the protective effects of *M. communis* against acute and chronic inflammation using xylene-induced ear edema model and the cotton pellet-induced granuloma test in mice, respectively. Aqueous and ethanolic extracts of the aerial parts of myrtle were administered intraperitoneally to albino mice prior to inducing ear edema with xylene in the acute inflammation model. Intraperitoneal administration of the extracts was performed daily for 7 days to mice bearing subcutaneously implanted cotton pellets to induce chronic inflammation. While the aqueous extract exhibited significant anti-inflammatory activity in a dose-dependent manner, the ethanolic extract was effective in suppressing both acute and chronic inflammation across all administered doses (Hosseinzadeh et al. 2011).

Myeloperoxidase (MPO) is a peroxidase enzyme released by activated neutrophils and serves as a common biomarker of inflammation (Hanning et al. 2023); therefore, a reduction in MPO levels is indicative of the anti-inflammatory effect of the administered agent. In a study using a croton oil-induced ear edema model in mice, topical application of the EO obtained by hydrodistillation of *M. communis* leaves significantly reduced ear edema at 6 h and decreased MPO activity in tissue homogenates at 24 h in a dose-dependent manner. Moreover, in a cotton pellet-induced granuloma model in Wistar albino rats, topical application of myrtle EO for 10 days resulted in a significant decrease in granuloma formation, as well as in serum levels of TNF-α and IL-6 (Maxia et al. 2011). Altogether, the findings suggest that the anti-inflammatory effects of *M. communis* might be mediated by the suppression of pro-inflammatory cytokines.

More recently, Mechchate et al. investigated the anti-inflammatory and healing effects of a methanolic polyphenol-enriched fraction (PEMC) obtained from *M. communis* leaves*.* Wistar rats were pretreated with a polyphenol-enriched myrtle extract via oral gavage, prior to the induction of carrageenan-induced paw edema. Administration of PEMC produced a dose-dependent reduction in paw edema after 6 h, showing an efficacy comparable to diclofenac, a conventional anti-inflammatory agent (Mechchate et al. 2022). Consistently, studies by Belahcene et al. demonstrated that orogastric administration of both methanolic extract and EO of *M. communis* leaves exerted an anti-inflammatory effect on carrageenan-induced paw edema in Wistar albino rats within 4 h, comparable to diclofenac (Belahcene et al. 2023, 2024), thereby supporting the potential use of *M. communis* as an alternative anti-edema and anti-inflammatory agent.

#### Burn and wound healing models

*M. communis* has long been used in folk medicine for burn and wound healing, paving the way for in vivo studies employing corresponding animal models. In a study investigating the wound-healing effects of *M. communis*, methanolic extracts of myrtle stems and seeds were tested on oral ulcers in Sprague–Dawley rats, in comparison with extracts of *Camellia sinensis* L. (leaves) and *Zataria multiflora* Boiss. (leaves and stems). After applying the extracts as mucosa patches on the wounds for 8 days, tissue inflammation was evaluated as a histopathological criterion. Although all plant extracts were efficient on ameliorating the parameters associated with oral wound healing, *M. communis* exhibited the most pronounced effect, leading to decreased inflammation scores and neutrophil counts, along with an increase in fibroblast numbers (Hashemipour et al. 2017).

Thermal skin burns trigger acute systemic inflammation, characterized by an imbalance between oxidative and antioxidative components that also affect inflammatory mediators such as NO. In an experiment evaluating the effects of *M. communis* on burn-induced skin damage, the ethanol extract of myrtle leaves was administered twice daily for 2 days, either orally or topically as an ointment applied to the dorsal wounds of Wistar Albino rats. A burn-induced reduction observed in skin NO levels was restored solely by oral administration of myrtle extract. Histological analysis revealed that topical application of myrtle extracts partially reduced inflammation, characterized by moderate leukocyte accumulation and vascular congestion, compared with the severe inflammation and tissue damage observed in the burn group. Oral treatment was more effective in alleviating these inflammatory changes. Although neither application completely prevented tissue damage, the findings suggested that *M. communis* may serve as a potential treatment agent for burn-induced injury due to its accelerating effect in tissue factors which play a role in coagulation (Ozcan et al. 2019).

The wound-healing properties of *M. communis* were also evaluated on heat-induced burn wounds infected with methicillin-resistant *Staphylococcus aureus* (MRSA) in Wistar rats. Ointments containing methanolic extracts of myrtle leaves were applied to the wound surfaces, either alone or in combination with silver nanoparticles, for 14 days, in comparison with silver sulfadiazine and mupirocin ointments. Notably, the myrtle extract alone produced the greatest reduction in burn area, followed by its combination with silver nanoparticles, which demonstrated the second most effective outcome among all treatments. In contrast to the findings of Ozcan et al. (2019), where burn induction led to decreased skin NO levels, serum NO levels were elevated in rats with infected burn wounds. The application of myrtle extract, as well as silver nanoparticle and mupirocin ointments, efficiently reduced NO levels, suggesting that *M. communis* could be used to treat burn wound infections caused by MRSA due to its anti-inflammatory, wound healing, and antimicrobial properties (Jafari et al. 2024).

Recently, the regenerative potential of *M. communis* for deep burn injury was investigated in albino rats. A topical paste containing myrtle extract was applied for 21 days, either alone or in combination with intradermal mesenchymal stem cell injection. After the treatment, histological examination of skin samples and the expression levels of TNF-α and IL-6 were evaluated. Application of mesenchymal stem cells, either alone or in combination with myrtle extract, promoted skin regeneration and suppressed TNF-α and IL-6 expression, indicating that the anti-inflammatory effect of stem cells is greater than that of *M. communis* extract alone (Sarwar et al. 2025).

#### Organ-specific models

##### Gastrointestinal system models

Most studies investigating the anti-inflammatory effects of *M. communis* have focused on leaf extracts, whereas research on its berries or seeds remains limited. In the study by Jabri et al., the effects of aqueous extracts of myrtle seeds on reflux-induced esophagitis were investigated in Wistar rats. Oral administration of the extracts for 6 h was observed to prevent morphological and histopathological alterations in the esophageal mucosa in a dose-dependent manner, exhibiting an efficacy comparable to that of famotidine and gallic acid (Jabri et al. 2016).

Despite their crucial roles in mediating inflammatory responses, only a limited number of studies on *M. communis* have incorporated cytokine measurements. In a study exploring the protective effects of myrtle in acute pancreatitis, levels of the pro-inflammatory cytokines IL-1β and IL-6, as well as the anti-inflammatory cytokine IL-10, were measured in Wistar albino rats. Myrtle leaf extracts were administered orally to the rats for 14 days prior to the induction of acute pancreatitis. The extract prevented the pancreatitis-related increases in IL-1β and IL-6 levels and suppressed the concurrent elevation in MPO activity. Conversely, the reduction in IL-10 levels associated with acute pancreatitis was reversed by myrtle extract treatment. The chemiluminescence values of luminol and lucigenin, which reflect phagocyte-derived activity in inflammatory processes (Tseng and Kung 2013) and is increased during pancreatitis, was significantly reduced by treatment with the extract. Additionally, myrtle extract prevented pancreatic edema and inflammatory cell infiltration, further supporting its protective role against inflammation (Ozbeyli et al. 2020).

Another study utilizing myrtle seeds investigated their anti-inflammatory and ROS-scavenging activities in Swiss albino mice infected with the gastrointestinal nematode *Heligmosomoides polygyrus*. Aqueous extracts of myrtle berry seeds were administered orally to the mice daily from days 18 to 21 of infection and their effects were compared with standard albendazole treatment. Administration of the extract significantly reduced the elevated serum levels of IL-1β and IL-6 in infected animals, while also decreasing adult worm counts and normalizing intestinal ROS levels. Histopathological examination of small intestinal tissues revealed that inflammatory cell infiltration in the infected group was reduced by treatment with either myrtle extract or albendazole, resulting in better preservation of the intestinal mucosa (Jabri et al. 2021).

The myrtle leaf extracts were also tested on pancreas of rats fed a high-fat diet (HFD). Following 4 months of orogastric gavage administration of ethanolic *M. communis* leaf extracts to Wistar albino rats, the elevated MPO levels and oxidative damage markers in pancreatic tissues of HFD-fed rats were ameliorated. Moreover, immunohistochemical staining revealed that the elevated levels of NF-κB and alpha-smooth muscle actin (α-SMA), an indicator of myofibroblast activation in chronic inflammation, were normalized following extract treatment. Histopathological analyses further supported the findings that myrtle extract alleviated pancreatic tissue damage through its anti-inflammatory and antioxidant mechanisms (Kabatas et al. 2024).

The in vivo gastroprotective potential of *M. communis* against HCl/EtOH-induced acute gastric lesions was evaluated by Mansour et al. Wistar rats were orally administered increasing doses of microencapsulated EOs, obtained by distillation from myrtle leaves, for 21 days prior to the induction of acute gastric ulcer. Pretreatment with the EOs exhibited a pronounced anti-inflammatory effect on the gastric mucosa, characterized by dose-dependent reduction of submucosal edema and inflammatory cell infiltration, as well as significant suppression of gastric lesion formation and acidity. Furthermore, elevated NO levels in tissue homogenates were significantly decreased at all administered doses of myrtle EOs (Mansour et al. 2022).

##### Hepatic and renal models

In an investigation of the antifibrotic and antioxidant properties of *M. communis* on liver injury and fibrosis in bile duct-ligated (BDL) Wistar albino rats, inflammatory parameters were also evaluated following oral administration of ethanol extracts of the myrtle leaves. Treatment with the myrtle extract for 28 days resulted in a marked reduction in the elevated serum levels of the pro-inflammatory cytokines TNF-α and IL-1β in BDL rats. The increases in MPO activity and TGF-β levels observed in the BDL group were significantly diminished in the extract-treated group. Moreover, the elevated chemiluminescence values of luminol and lucigenin in the liver tissues of the BDL group were significantly decreased following administration of the myrtle extract. Together with the microscopic observations, it was concluded that administration of *M. communis* extract protects liver tissue against oxidative damage by inhibiting neutrophil infiltration (Sen et al. 2016b).

Despite extensive research on the leaves of* M. communis*, there is a notable lack of studies addressing the anti-inflammatory effects of its flowers. Ben Hsouna et al. evaluated the EO obtained by hydrodistillation of *M. communis* flowers in a CCl_4_-induced hepatotoxicity model in Wistar rats. Intraperitoneal administration of the EOs for 14 days significantly attenuated oxidative stress imbalance and neutrophil infiltration in rats with CCl_4_-induced hepatotoxicity (Ben Hsouna et al. 2019).

Over the past decade, in vivo studies exploring the potential of *M. communis* to prevent or ameliorate oxidative organ damage, which is often accompanied by inflammatory processes, have gained increasing attention. Recently, the protective and therapeutic effects of myrtle leaves were examined in nephrolithiasis, a kidney disorder associated with oxidative damage. Ethanol extracts of myrtle leaves were orally administered to two groups of Wistar albino rats. In the prophylactic group, myrtle extract was given daily for 8 weeks concurrently with ethylene glycol (EG), a chemical agent used to induce nephrolithiasis. In the treatment group, rats were exposed to EG for 8 weeks, followed by myrtle extract administration during the final 4 weeks. In both groups, oral administration of myrtle extract reversed the nephrolithiasis-induced increases in MPO levels, as well as the biomarkers of oxidative damage, apoptosis, and cellular necrosis in renal tissues, consistent with histological findings. In addition, EG exposure elevated the levels of osteopontin, a molecule produced by various immune cells and implicated in multiple chronic inflammatory and autoimmune diseases (Kahles et al. 2014). While prophylactic administration of the extract effectively lowered osteopontin levels, therapeutic treatment was found to be inadequate. However, when considered collectively, the findings suggested that myrtle extract may mitigate histopathological alterations and oxidative stress in kidney tissues, functioning as either a preventive or therapeutic agent (Ertas et al. 2024).

##### Central nervous system (CNS) models

In addition to its protective and therapeutic roles in the gastrointestinal, hepatic, and renal systems, *M. communis* has also demonstrated neuroprotective and anti-inflammatory effects in the central nervous system (CNS), in a limited number of studies. Neuro-inflammation and oxidative stress are key pathological features of Alzheimer’s disease (AD). Although acetylcholinesterase (AChE) inhibitors, primarily galantamine (GAL), are currently used in AD treatment, their efficacy diminishes as the disease progresses, rendering them insufficient as long-term therapy. To date, no effective long-term treatment exists that can cure or modify the progression of AD; however, anti-inflammatory and antioxidant agents have been employed as supportive adjuvant therapies (Kumar and Singh 2015). In the study by Aykac et al., the protective effects of *M. communis* against AD were evaluated by orally administering ethanol extract of myrtle leaves to scopolamine-induced Wistar albino rats, an AD model, for 14 days, in comparison with GAL. Myrtle treatment significantly reduced neutrophil infiltration and the associated MPO levels in AD brain tissue, thereby suppressing inflammation. Overall, *M. communis* was shown to be a potential regulatory agent in AD treatment, exhibiting comparable efficacy to GAL in modulating inflammation, while also improving cognitive function and regulating cholinergic activity, neurotransmitter levels, and antioxidant defenses (Aykac et al. 2019).

The ameliorative effects of *M. communis* on cognitive impairment were also investigated in a renovascular hypertensive rat model, with ramipril serving as a reference treatment. Oral administration of ethanol extract of myrtle leaves to Wistar albino rats for 9 weeks improved cognitive function and exerted anti-inflammatory effects. While myrtle extract administration reduced the elevated osteopontin levels in hypertensive rats, it also significantly restored the decreased serum IL-10 levels, an indication of impaired anti-inflammatory activity. Moreover, levels of metalloproteinase (MMP)-13 and CD36, which were increased in hypertensive rats and associated with neuroinflammation, were diminished following *M. communis* treatment. Similarly, elevated amyloid beta (Aβ) deposition and AChE levels in brain tissue, both directly associated to neuroinflammation and neurodegeneration, were suppressed by myrtle extract treatment. Collectively, these findings indicated that *M. communis*, with efficacy comparable to ramipril, suppresses the hippocampal inflammation and attenuates neurodegenerative processes (Cevikelli-Yakut et al. 2020).

In addition to its anti-helminthic properties, the anti-parasitic effect of *M. communis* was also investigated in mice infected with *Toxoplasma gondii*. Toxoplasmosis, caused by *Toxoplasma gondii,* primarily affects the CNS, particularly the brain and retina (Wohlfert et al. 2017). To investigate the prophylactic effects of myrtle against chronic toxoplasmosis, BALB/c mice infected with *Toxoplasma gondii* received oral administration of EOs distilled from *M. communis* leaves at increasing concentrations. After a 3-week treatment, cysts numbers decreased, whereas IL-12 and IFN-γ mRNA levels increased in brain tissues, in line with the administered extract dose (Shaapan et al. 2021). According to the findings, the anti-parasitic effect of *M. communis* may be associated with its ability to enhance innate immune responses, as reflected by increased pro-inflammatory cytokine levels, highlighting its potential to modulate inflammatory processes rather than directly exert anti-inflammatory effects.

##### Other organ models

Methanolic macerates of *M. communis* leaves were administered intraperitoneally to albino rats for 14 days to examine their protective effects against pulmonary fibrosis. Both preventive and therapeutic administration of the extracts inhibited parenchymal inflammation in rats with pulmonary fibrosis (Samareh Fekri et al. 2018).

In a comparative study of *M. communis* and *Laurus nobilis* L. (laurel), EOs obtained by hydrodistillation from the leaves of both plants were administered intragastrically to Sprague Dawley rats for 14 days to evaluate their antioxidant and anti-atherogenic effects. Treatment with laurel and myrtle EOs led to an elevation in β-glucuronidase activity—an important bacterial enzyme associated with inflammatory processes—in the intestinal contents of rats. However, administration of both EOs resulted in epithelial alterations in the ileum and disruption of villous and submucosal structural integrity. Remarkably, myrtle administration caused more pronounced damage to intestinal tissue compared with laurel. Histological examination following myrtle treatment revealed inflammatory cell infiltrates including neutrophils, macrophages, eosinophils, and lymphocytes within the intestinal crypts, indicative of active inflammation. Moreover, myrtle administration reduced the number of probiotic bacteria while increasing Enterobacter levels, thereby promoting intestinal inflammation. The increase in relative liver and lung weights induced by myrtle EO further supported its pro-inflammatory potential. In contrast to most studies reporting anti-inflammatory effects of *M. communis*, the findings reported that myrtle EO may exert tissue-dependent pro-oxidative effects in addition to its antioxidant potential, underscoring the need for careful assessment of tissue-specific responses and optimization of dosing in therapeutic applications (Odeh et al. 2022).

*M. communis* was also demonstrated to attenuate HFD-induced testicular damage in rats by reducing oxidative stress. Wistar albino rats were fed an HFD for 16 weeks, and ethanol extracts of myrtle leaves were administered orally during the final 4 weeks. The extract reversed the HFD-induced increase in MPO levels and oxidative damage markers, and enhanced cellular antioxidant capacity in testicular tissues, indicating a potential anti-inflammatory effect of *M. communis* against HFD-induced testicular damage (Coskunlu et al. 2024).

### Clinical trials

#### Aphthous stomatitis and gingivitis

The number of clinical trials on *M. communis* is considerably fewer than in vivo and in vitro studies, with only a limited number available. The existing clinical studies on the anti-inflammatory properties of *M. communis* have primarily focused on conditions such as aphthous stomatitis, gingivitis, and acne vulgaris. In one of the earliest clinical studies by Babaee et al., myrtle extracts were applied to recurrent aphthous stomatitis, a painful and ulcerative condition of the oral cavity. An oral paste containing a boiling water extract of myrtle leaves, along with a placebo paste replacing the classical treatment, was applied topically for 6 days to patients with recurrent aphthous stomatitis. Significant differences in ulcer size and pain intensity were observed between the experimental and placebo groups throughout the treatment period. Moreover, the myrtle oral paste-treated group showed markedly reduced erythema and exudation relative to the placebo group, with overall significant improvements observed across all evaluated clinical parameters (Babaee et al. 2010).

Subsequent clinical investigations into the anti-inflammatory effects of *M. communis* remained notably scarce for an extended period, though interest has grown in recent years. In a cross-over clinical trial, an herbal mouthwash containing ethanolic extracts of 5 plants, including *M. communis, Quercus brantii*, *Punica granatum*, *Portulaca oleracea,* and *Boswellia serrata*, was evaluated for its efficacy against gingivitis. Patients with plaque-induced gingivitis were divided into two groups and treated with either the herbal or chlorhexidine mouthwash twice daily for 2 weeks, after which the treatments were crossed over for an additional 2 weeks. Post-treatment evaluation of clinical parameters revealed no significant differences between the two groups, indicating that both mouthwashes exhibited comparable therapeutic effects and suggesting that the herbal mouthwash containing *M. communis* extract could be considered an alternative treatment for plaque-induced gingivitis (Talebi Ardakani et al. 2022).

Recently, a similar study assessed the efficacy of mouthwashes containing ethanolic or aqueous extracts of a mixture of *Populus euphratica* and *M. communis* leaves in pediatric patients with gingivitis, in comparison with chlorhexidine and placebo mouthwashes. After 2 weeks of administration, the mouthwashes were shown to significantly reduce gingivitis, suggesting their potential applicability in pediatric patients (Mohamed-Ali et al. 2024).

#### Acne vulgaris

The clinical trials assessing the inflammation-modulating activity of *M. communis* have been conducted predominantly within the last 5 years. Building on in vitro research, clinical studies have examined the application of *M. communis* in the treatment of acne vulgaris. In a split-face study, a topical solution containing an ethanolic extract of *M. communis* leaves was applied to one side of the face, while a clindamycin solution was applied to the other side for 12 weeks. By the end of the treatment, both groups showed significant reductions in inflammatory lesions and the acne severity index; however, the improvement was more pronounced in the group treated with myrtle solution. The findings indicated that myrtle is an effective and safe treatment for mild to moderate acne vulgaris, demonstrating enhanced anti-inflammatory activity (Salmanian et al. 2020).

In another study on acne vulgaris, a topical product containing *M. communis* extract and azelaic acid was applied to patients’ faces for 16 weeks and compared with a light moisturizing cream. After completion of the treatment period, acne recurrence rates were significantly lower in the group treated with the myrtle extract-containing product than in the control group. However, neither product led to a significant reduction in the number of inflammatory acne lesions, and no significant differences were observed between the groups. Although these results support the potential therapeutic role of *M. communis* in acne management, they do not provide evidence for its anti-inflammatory activity (Bagatin et al. 2023).

Lastly, Mias et al. investigated a dermocosmetic product containing standardized *M. communis* leaf extract and CEE extracts by testing its activity against *C. acnes* and assessing clinical parameters in patients with mild to moderate acne vulgaris. After 57 days of treatment, a progressive decrease was observed in all lesion types, including inflammatory lesions and overall acne severity (Mias et al. 2023b). Although the anti-inflammatory potential of the product cannot be attributed solely to *M. communis*, its presence in combination with celastrol in the formulation may have contributed to the reduction in acne severity.

## Discussion

In vitro studies on the anti-inflammatory potential of *M. communis* have been conducted over the past decade, with approximately half focusing on immune cells and the remainder on other cell types. In contrast to in vivo experiments, myrtle fruits have been more frequently used in cell-based studies. In investigations involving immune cells, the effects of *M. communis* have been examined in human peripheral blood neutrophils, monocytes and dendritic cells, as well as mouse mast cells and macrophages. It has been shown that myrtle fruit extracts may contribute to the suppression of inflammatory responses by inducing neutrophil apoptosis and inhibiting mast cell degranulation. In addition, MCA-1 and myrtle EO have been found to suppress molecules involved in inflammatory processes in human monocytes and mouse macrophages. Only one of these studies measured cytokine production (IL-6, IL-12p40, TNF-α, and IL-8) in human Mo-DCs and reported their decreased levels following exposure to myrtle leaf extracts, particularly when used in combination (Mias et al. 2023a).

In none-immune cell models, gastric epithelial cells, human keratinocytes, skin fibroblasts, and umbilical cord vein endothelial cells were used to determine the inflammation-suppressing activity of *M. communis*. In these studies, *M. communis* fruit extracts consistently suppressed key pro-inflammatory cytokines (IL-1α, IL-1β, IL-8 and TNF-α) in infection-induced and oxidative stress-mediated inflammatory settings. However, the observed effects in LPS-induced in vitro model of endothelial cell damage in HUVECs, the anti-inflammatory activity of *M. communis* in endothelial cells appeared to be compound-dependent rather than a general property of the myrtle EO (Kutlu et al. 2021). The predominant use of myrtle fruit extracts in in vitro studies provides supportive evidence for their anti-inflammatory effects; however, it also reveals a critical lack of information regarding the effects of leaf extracts of *M. communis*. Moreover, the variability in experimental parameters, including the cell types and inflammatory stimuli employed across studies, complicates the establishment of standardized conclusions regarding the in vitro effects of myrtle fruits.

The majority of the available studies with the potential use of *M. communis* in the management of inflammation have mainly been performed in animal models. Nevertheless, the use of plant parts other than the leaves has remained quite limited, and the anti-inflammatory effects have been mainly evaluated through clinical assessments and biochemical parameters. In these studies, the anti-edematous, wound- and burn-healing, and organ-specific anti-inflammatory effects of myrtle extracts or EOs were evaluated following administration to animals via oral, intraperitoneal, or topical routes. Regardless of the route of administration, the extracts and EO obtained from myrtle were shown to possess both anti-edematous and anti-inflammatory activities.

Findings from animal models indicate that *M. communis* possesses significant wound- and burn-healing potential, primarily attributed to its anti-inflammatory, antioxidant, and antimicrobial activities. Ethanol or methanol extracts of *M. communis*, applied either orally or topically, have been shown to accelerate tissue repair, modulate inflammatory responses, and restore oxidative balance. Although these effects occur independently from the administration route, evidence from the study by Ozcan et al. suggests that oral administration of myrtle extracts may more effectively modulate tissue-level alterations in burn injuries compared with topical application (Ozcan et al. 2019).

In gastrointestinal models, extracts obtained from *M. communis* leaves and seeds were administered orally. Both short- and long-term oral administration of myrtle extracts and EOs have been demonstrated to present protective effects in experimental inflammatory models of the gastrointestinal organs by attenuating inflammatory responses. The consistent protective outcomes across different gastrointestinal inflammation models suggest that orally delivered *M. communis* exerts both local and systemic anti-inflammatory effects.

In addition to the oral administration of ethanol extracts of myrtle leaves, EO obtained from *M. communis* flowers was administered intraperitoneally in models of chronic liver and renal injury. Whether applied prophylactically or therapeutically, *M. communis* has been shown to play anti-inflammatory effects on liver and renal tissues by attenuating oxidative damage and inhibiting neutrophil infiltration.

In recent years, studies investigating the effects of *M. communis* on the inflammatory and neurodegenerative disorders of the CNS have been conducted, employing orally administered EOs and ethanol extracts from myrtle leaves. The findings showed that *M. communis* exerts ameliorative effects on neuroinflammation and neurodegeneration, with evidence suggesting that these effects are mediated through the suppression of neutrophil infiltration and pro-inflammatory cytokines in brain tissues.

Experimental animal studies have also investigated the therapeutic or protective potential of *M. communis* on the respiratory, metabolic/cardiovascular, and male reproductive systems. Extracts and EOs from myrtle leaves were administered intraperitoneally, intragastrically, or orally in models of pulmonary fibrosis, atherogenic and testicular damage, respectively. Regardless of whether administered prophylactically or therapeutically, myrtle leaf extracts have been shown to inhibit parenchymal inflammation in lungs and to exert potential anti-inflammatory effects on testicular tissues. However, unlike the vast majority of in vivo experiments, the study by Odeh et al. suggested that *M. communis* may elicit pro-inflammatory effects. In this comparative study, EOs obtained from the leaves of *M. communis* and *Laurus nobilis* were administered intragastrically to Sprague Dawley rats, and both plants—particularly *M. communis*—were found to induce intestinal tissue damage, inflammatory cell infiltration, and a reduction in probiotic bacteria, with findings indicating the development of inflammation in the intestinal tissues as well as in the liver and lungs (Libby 2007). The possibility that these results, which conflict with numerous studies demonstrating the anti-inflammatory effects of *M. communis*, may be attributable to methodological differences should not be overlooked.

Overall, as noted above, investigations examining the effects of *M. communis* on inflammation are predominantly based on histopathological evaluations, clinical analyses, and biochemical measurements. One important limitation is that the humoral and cellular components of the immune system, which play a central role in inflammatory processes, have not been adequately investigated. In studies that have incorporated the immune system, the focus has largely been on histopathological assessments of infiltration of pro-inflammatory cells such as neutrophils, while a lack of functional studies is evident. Regarding measurements of cytokines, which are key humoral components of the immune system and play an important role in inflammation, *M. communis* administration has been demonstrated to reduce serum and tissue levels of the pro-inflammatory cytokines IL-1β, IL-6, and TNF-α (Maxia et al. 2011; Ozbeyli et al. 2020; Sen et al. 2016b; Jabri et al. 2021; Sarwar et al. 2025). Consistently, there are findings of increased levels of IL-10, which is considered as an anti-inflammatory cytokine, both in serum and tissues, following oral myrtle leaf extract treatment (Ozbeyli et al. 2020; Cevikelli-Yakut et al. 2020). Although levels of IL-12 and IFN-γ were found to be increased by the administration of myrtle leaf EOs in brain tissues, the elevation of these pro-inflammatory cytokines was suggested to indicate that *M. communis* may modulate inflammatory processes by strengthening innate immune responses instead of possessing an exact anti-inflammatory activity (Shaapan et al. 2021).

The clinical use of *M. communis* has largely emerged only in the past few years. These studies have primarily focused on oral applications of the plant in conditions such as aphthous stomatitis and gingivitis, as well as its topical use in acne vulgaris. Topical intraoral applications of *M. communis* leaves, either alone or in combination, demonstrated comparable healing effects on aphthous stomatitis and gingivitis relative to both standard treatment and placebo, depending on the clinical observations. Topical applications of *M. communis* on acne vulgaris, either alone or in combination with other agents, have been shown to significantly reduce acne severity and lesions, with effects comparable to or even greater than standard treatment. Although Bagatin et al. did not observe a significant reduction in inflammatory acne lesions, they reported that the application of *M. communis* extract led to a greater decrease in acne recurrence rates compared to the control group (Bagatin et al. 2023). Collectively, these studies provide strong evidence supporting the potential use of *M. communis* as an effective agent in the treatment of acne vulgaris.

However, it is noteworthy that in clinical studies addressing both aphthous stomatitis and gingivitis as well as acne vulgaris, the effects of *M. communis* have been assessed solely based on clinical observations, without evaluation of biochemical or immunological parameters. Moreover, the exclusive use of the plant’s leaves in clinical studies, coupled with the lack of investigations involving the fruits or flowers, highlights another gap in literature. Considering its widespread use in traditional medicine, expanding clinical investigations on different parts of the plant may facilitate the development and eventual therapeutic application of standardized pharmaceutical formulations.

## Conclusion

This review provides a comprehensive perspective on the anti-inflammatory effects of *M. communis* and highlights that most of in vivo studies occupying a significant portion of the literature have focused on the leaves of the plant. Although the use of fruits is more frequent in in vitro studies than in animal experiments, it is evident that further investigations are needed to evaluate the effects of the non-leaf parts of *M. communis* on inflammatory parameters, especially in immune cell-based models. Overall, despite consistent evidence supporting the anti-inflammatory potential of *M. communis* across in vitro, in vivo, and clinical studies, important mechanistic and translational gaps remain. The expansion of standardized studies incorporating both humoral and cellular components of the immune system, which are key regulators of inflammatory processes, is essential to clarify the mechanisms underlying the largely clinical observation-based anti-inflammatory effects of *M. communis*.

With few exceptions, evidence from experimental and clinical studies consistently supports the anti-inflammatory properties of *M. communis*. However, further research is required to standardize extraction methods, concentrations, routes of administration, and treatment duration to ensure its safe and effective use as an alternative anti-inflammatory therapeutic and/or prophylactic agent. In addition, building on findings from animal models, the expansion of well-designed clinical trials across a broader range of inflammatory conditions is crucial for the scientific validation of the traditional uses of *M. communis*. Finally, immune system–based studies may further elucidate its systemic mechanisms of action and support the development of targeted pharmaceutical formulations.

## Data Availability

No datasets were generated or analysed during the current study.
